# Among overweight middle-aged men, first-borns have lower insulin sensitivity than second-borns

**DOI:** 10.1038/srep03906

**Published:** 2014-02-06

**Authors:** Benjamin B. Albert, Martin de Bock, José G. B. Derraik, Christine M. Brennan, Janene B. Biggs, Paul L. Hofman, Wayne S. Cutfield

**Affiliations:** 1 Liggins Institute, University of Auckland, Auckland, New Zealand; 2 Gravida: National Centre for Growth and Development, University of Auckland, Auckland, New Zealand

## Abstract

We aimed to assess whether birth order affects metabolism and body composition in overweight middle-aged men. We studied 50 men aged 45.6 ± 5.5 years, who were overweight (BMI 27.5 ± 1.7 kg/m^2^) but otherwise healthy in Auckland, New Zealand. These included 26 first-borns and 24 second-borns. Insulin sensitivity was assessed by the Matsuda method from an oral glucose tolerance test. Other assessments included DXA-derived body composition, lipid profiles, 24-hour ambulatory blood pressure, and carotid intima-media thickness. First-born men were 6.9 kg heavier (p = 0.013) and had greater BMI (29.1 vs 27.5 kg/m^2^; p = 0.004) than second-borns. Insulin sensitivity in first-born men was 33% lower than in second-borns (4.38 vs 6.51; p = 0.014), despite adjustment for fat mass. There were no significant differences in ambulatory blood pressure, lipid profile or carotid intima-media thickness between first- and second-borns. Thus, first-born adults may be at a greater risk of metabolic and cardiovascular diseases.

There has been a steady reduction in birth rates throughout the world^1^. Therefore, average family size has decreased, with a consequent increase in the proportion of first-born children in many countries^2^. Thus, any adverse health outcomes associated with being first-born would affect an increasing proportion of the world's population^3^.

There is some evidence that birth order influences growth and metabolism, from infancy to early adulthood^3^. First-born babies have lower birth weight, but more rapid growth and weight gain in infancy^4^,^5^, such that in childhood they are taller than later-borns^2^,^6^. Importantly, first-born children have reduced insulin sensitivity and higher daytime blood pressure^2^. Although the height discrepancy is reduced by early adulthood, first-borns have greater adiposity^4^,^7^. Further, first-borns have been shown to have a less favourable lipid profile in young adulthood, with higher LDL-C, total cholesterol, and triglyceride concentrations than later-borns^4^. Thus, being first-born may be associated with persistent changes in metabolism and body composition, that may lead to greater risk of developing type 2 diabetes mellitus and cardiovascular disease.

While there are limited data on the effects of birth order on growth and metabolism in the first two decades of life, there are no data on such outcomes in mid-adulthood. It is important to assess whether the metabolic effects observed in early years are sustained or magnified throughout adulthood, particularly since middle age is a time where identification of risk factors and early intervention may be most appropriate. Therefore, we aimed to assess whether birth order affects metabolism and body composition in overweight middle-aged men.

## Results

**Figure 1:**
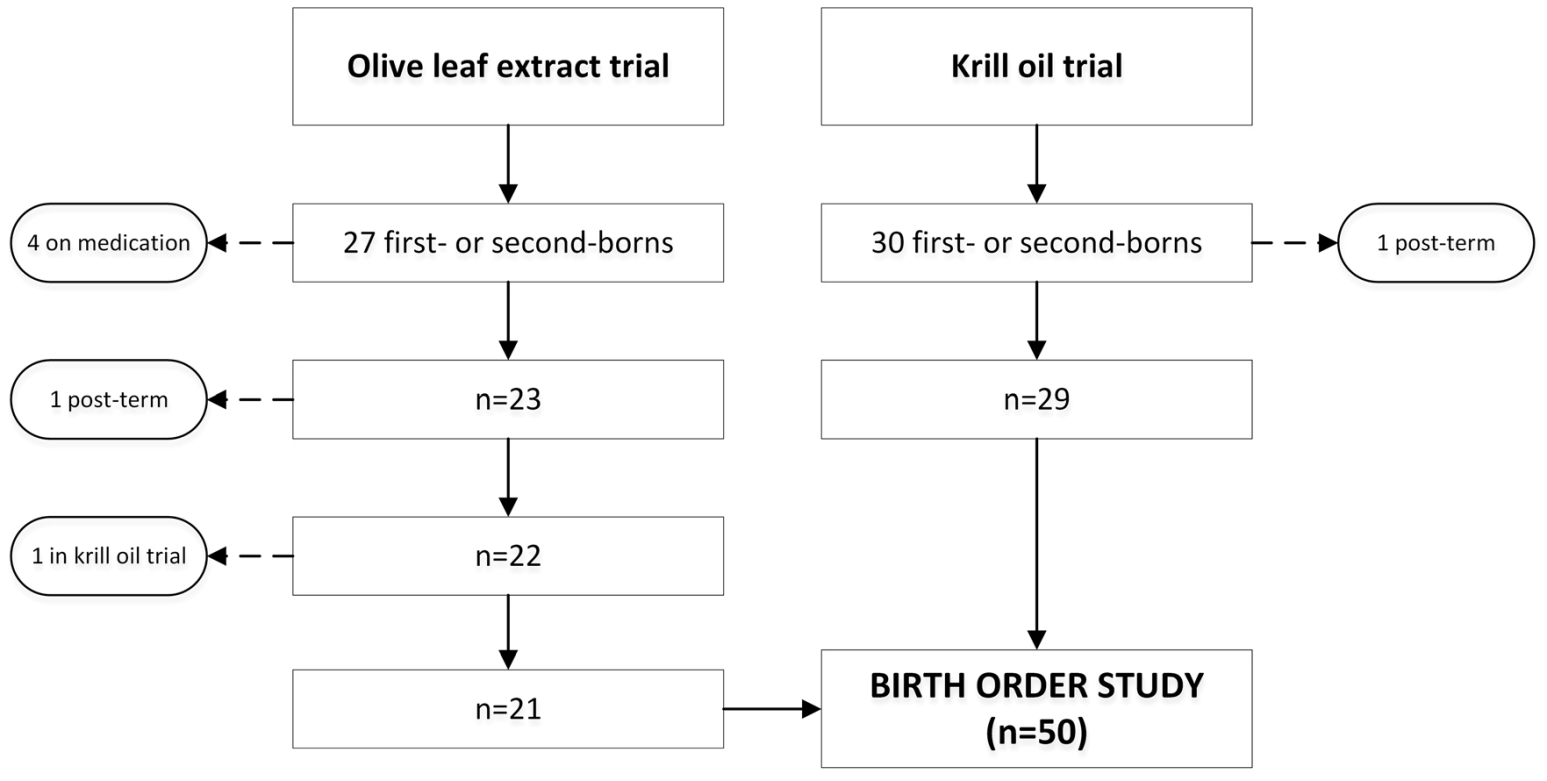
Summary of study recruitment. All participants from the olive leaf extract^8^ and krill oil (unpublished data) trials were overweight middle-aged men recruited in Auckland, New Zealand.

From the 57 first- and second-born men that participated in both trials, 50 subjects met the inclusion criteria (Figure 1). Participants were aged 45.6 ± 5.5 years and of BMI 27.5 ± 1.7 kg/m^2^, including 26 first-borns and 24 second-borns. The vast majority of participants (92%) were of European descent, and men in both groups were of similar age and ethnicity (Table 1).

First- and second-born men were of similar height (p = 0.40; Table 1). However, first-born men were 6.9 kg heavier (p = 0.013) and had greater BMI (29.1 vs 27.5 kg/m^2^; p = 0.004) than second-borns (Table 1). There were no significant differences between groups in total body fat and android fat to gynoid fat ratio, but first-borns tended to have a greater fat mass index than second-borns (p = 0.068; Table 1).

Insulin sensitivity in first-born men was 33% lower than in second-borns (4.38 vs 6.51; p = 0.014) (Table 2), despite adjustment for confounders including fat mass, socio-economic status, and physical activity levels. However, disposition index was similar in both groups (Table 2). Further, there were no significant differences in ambulatory blood pressure, lipid profile, or carotid intima-media thickness between first- and second-borns (Table 2).

## Discussion

This study has shown that within a cohort of overweight middle-aged men, first-borns had greater BMI and lower insulin sensitivity than second-borns. Importantly, the difference in insulin sensitivity was independent of fat mass. Reduced insulin sensitivity is an independent predictor of type 2 diabetes mellitus, hypertension, coronary heart disease, stroke and cancer in non-obese middle aged men^15^.

Recently, there has been increasing interest in two atypical groups. Firstly, the metabolically healthy obese (MHO)^16^, who although obese do not display the typical adverse metabolic effects of obesity. Secondly, the metabolically obese but normal weight (MONW)^16^, who have unhealthy metabolic markers despite being in the normal BMI range. Environmental^17^ and genetic^18^ correlates of MHO have been identified. We speculate that birth order, through an influence on the early fetal environment, contributes to the phenotype of insulin resistance and metabolic adversity that characterise the unhealthy obese and MONW.

The mechanisms by which birth order influences long-term metabolism are unknown, but differences in placental blood flow may play a role. During pregnancy, structural changes occur to uterine spiral arteries to facilitate placentation^19^. Following parturition, these changes do not reverse, suggesting a more favourable fetal environment for subsequent pregnancies^20^. Not surprisingly, first-borns are lighter at birth than later-borns^2^,^6^ Nonetheless, while placental blood flow is an important regulator of fetal growth^21^, the metabolic effect of birth order is not solely due to differences in birth weight^2^.

It is worth noting that the difference in insulin sensitivity was also independent of the age of participants and of their parents. Recent studies indicate that there are age-related changes in the β-adrenergic system that lead to altered cardiovascular function^22^ and reduced insulin secretion^23^. These observations highlight the importance of accounting for both parental and participants' ages in our analyses. Age-related changes could directly influence the metabolic phenotype of participants, while those born of older mothers could theoretically be affected via altered fetal growth and subsequent metabolic programming^24^.

The major strength of this study is the comprehensive metabolic assessment. Although the study of a narrow BMI range enabled characterization of overweight middle-aged men, this may limit wider application of our findings. Given first-borns had greater BMI than second-borns, using BMI as an inclusion criterion for the parent clinical trials might have introduced a subtle selection bias. Other limitations include the *post hoc* analysis and a relatively small sample size. In addition, we studied a relatively narrow range of individuals (overweight males living in a large urban centre, mostly of New Zealand European ethnicity), which could also limit the applicability to the general population, particularly females. Lastly, as we did not study sibling pairs, our findings could have underestimated the magnitude of birth order effects on insulin sensitivity and other metabolic outcomes.

In conclusion, first-born overweight middle-aged men have greater BMI and lower insulin sensitivity than second-borns. Larger studies are required to better evaluate the long-term health effects of birth order across the BMI range. Ideally, future research should focus on sibling pairs.

## Methods

### Ethics approval

Ethics approval was provided by the Central and Northern Y Regional Ethics Committees (Ministry of Health, New Zealand), and written informed consent was obtained from all participants.

### Participants & recruitment

Participants were recruited for two clinical trials investigating the metabolic effects of supplementation with olive leaf extract^8^ or krill oil (unpublished data) (Figure 1). Volunteers were recruited in 2011 and 2012 using advertisements in local newspapers that circulate freely in the central Auckland metropolitan area (New Zealand). First- and second-born men who were overweight (body mass index (BMI) 25–30 kg/m^2^) and middle-aged (35–55 years) were eligible to participate. Note that only males were recruited to the clinical trials so that the effects of the menstrual cycle and/or oral contraception on insulin sensitivity (the primary outcome) could be avoided. Exclusion criteria were: diabetes mellitus, hypertension (systolic blood pressure >145 mmHg or diastolic blood pressure >95 mmHg), known dyslipidaemia, or the use of tobacco, or prescription medications likely to affect blood pressure, lipid profile or insulin sensitivity. From this group, all participants born at term (37–41 weeks) from singleton pregnancies were included. Note that when assessing birth order, miscarriages of less than 20 weeks were not counted. Where subjects participated in more than one clinical trial, the data from the most recent trial was used.

### Clinical assessments

All clinical assessments were carried out at the Maurice & Agnes Paykel Clinical Research Unit (Liggins Institute, University of Auckland). Insulin sensitivity was assessed via a 75 g oral glucose tolerance test using the Matsuda method, with glucose and insulin samples collected at 0, 30, 60, 90, and 120 minutes^9^. The Matsuda index has a strong correlation with the hyperinsulinemic euglycaemic clamp (r = 0.77)^10^, and excellent reproducibility during multiple measures^11^. The oral disposition index (a measure of β-cell function corrected for insulin sensitivity) was also calculated.

Fasting blood samples were used to assess lipid profile, namely triglycerides, total cholesterol, high-density lipoprotein cholesterol (HDL-C), and low-density lipoprotein cholesterol (LDL-C). Auxological assessment included height measurement using a Harpenden stadiometer. Weight and body composition were assessed using whole-body dual-energy X-ray absorptiometry (DEXA, Lunar Prodigy 2000, General Electric, Madison, USA). Apart from BMI, the fat mass index was also calculated^12^.

24-hour ambulatory blood pressure monitoring was carried out prior to the clinical assessment. Participants were fitted with a Spacelabs 90207 or 90217 monitor (Spacelabs Medical Inc., Redmond, USA), with each subject being assigned the same device model for all assessments. Measurements were performed every 20 minutes between 07:00 and 22:00, and every 30 minutes from 22:00 to 07:00. Only profiles with >14 daytime and >7 night time recordings over a 24-hour period were analysed.

Carotid intima-media thickness (cIMT) was also measured, as it is a validated and reproducible measure that is predictive of cardiovascular and cerebrovascular risks. cIMT was measured using an M-Turbo ultrasound system (Sonosite, Bothel, USA) by trained investigators [MdB, BA], with longitudinal images attained using a standard protocol^13^. The right common carotid artery was scanned from both posterolateral and anterolateral views. Digitally stored images were analysed using computer software automated callipers to measure the far wall (SonoCalc^tm^ v.4.1, Sonosite). The maximal cIMT measurement from both views, approximately 10 mm proximal to the carotid bulb was used for comparative analysis. To assess reproducibility, triplicate measures were taken of seven healthy volunteers over a 7-day interval, and resulted in an intra-observer CV of 3.7% (unpublished data).

Physical activity levels were assessed using the International Physical Activity Questionnaire (IPAQ)^14^, covering four domains of physical activity: work-related, transportation, housework/gardening, and leisure time. Geo-coded deprivation scores were derived from current address using the New Zealand Index of Deprivation 2006 (NZDep2006). This index is based on household census data reflecting nine aspects of material and social deprivation to divide New Zealand into tenths (scored 1–10) by residential address. Scores of 1 represent the least deprived areas and 10 the most deprived. Scores are derived from units covering a small area, each reflecting approximately 90 people.

### Assays

Insulin concentrations were measured using an Abbott AxSYM system (Abbott Laboratories, Abbott Park, USA) by microparticle enzyme immunoassay with an inter-assay coefficient of variation (CV) of 5.4%. Glucose, triglyceride, total cholesterol, HDL-C, and LDL-C concentrations were measured on a Hitachi 902 autoanalyser (Hitachi High Technologies Corporation, Tokyo, Japan) by enzymatic colorimetric assay (Roche, Mannheim, Germany) with a CV lower than 2.5%. Highly sensitive CRP was measured using commercially available ELISA kits (OLE study, USCN Life Science, Wuhan, China, CV 10%) or a Hitachi 902 autoanalyser (Krill study, Hitachi High Technologies Corporation, CV 3.1%).

### Statistical analysis

Demographic characteristics between first- and second-born men were compared using one-way ANOVA and Fisher's exact tests in Minitab v.16 (Pennsylvania State University, State College, PA, USA). Multivariate linear regression models were carried out in SAS v.9.3 (SAS Institute, Cary, NC, USA). All models accounted for important confounding factors, namely age, ethnicity, socio-economic status (NZDep2006), physical activity levels (IPAQ), and mean parental age at childbirth. Age and total body fat percentage were also controlled for, when assessing potential differences in lipids and outcomes associated with glucose homeostasis. All statistical tests were two-tailed and significance level maintained at 5%. Demographic data are presented as means ± standard deviations, while other data are means and 95% confidence intervals adjusted for the confounders in multivariate models.

## Author Contributions

B.B.A., W.S.C., M.d.B., P.L.H. and J.G.B.D. conceived and designed the study. B.B.A., M.d.B., C.B. and J.B. recruited and performed the tests. B.B.A. and M.d.B. collected and compiled the data. J.G.B.D. carried out the statistical analyses. B.B.A., J.G.B.D. and W.S.C. wrote the manuscript with input from other authors. All authors have approved the submission of the final version of this manuscript.

## Figures and Tables

**Table 1:** Age and anthropometry in first-born and second-born men. Age data are mean ± SD; other data are means and 95% confidence intervals adjusted for other confounding factors in the multivariate models

	First-borns	Second-borns	p-value
**n**	26	24	
**Ethnicity (European descent)**	92%	92%	0.99
**Age (years)**	44.9 ± 5.6	46.4 ± 5.4	0.37
**Height (cm)**	176.8 (171.0–180.5)	175.1 (169.7–180.5)	0.40
**Weight (kg)**	91.0 (83.6–98.4)	84.1 (76.9–91.3)	0.013
**BMI (kg/m^2^)**	29.1 (27.7–30.5)	27.5 (26.1–28.9)	0.004
**Total body fat (%)**	32.2 (27.3–37.1)	29.9 (25.1–34.7)	0.21
**Android fat to gynoid fat ratio**	1.27 (1.13–1.41)	1.23 (1.09–1.36)	0.42
**Fat mass index (kg/m^2^)**	9.36 (7.74–10.97)	8.26 (6.69–9.84)	0.068

**Table 2:** Study outcomes in first-born and second-born men. Data are means and 95% confidence intervals adjusted for other confounding factors in the multivariate models

	First-borns	Second-borns	p-value
**n**	26	24	
**Glucose homeostasis**			
Insulin sensitivity (Matsuda index)	4.38 (2.72–6.73)	6.51 (4.31–9.56)	0.014
Disposition index	4.72 (4.65–4.79)	4.71 (4.64–4.78)	0.86
**24-hour ambulatory blood pressure**			
Systolic (mmHg)	128.3 (120.4–136.2)	124.7 (116.9–132.4)	0.23
Diastolic (mmHg)	80.6 (75.3–85.9)	77.9 (72.7–83.1)	0.18
Systolic dip (%)	13.5 (7.8–19.2)	12.8 (7.2–18.4)	0.76
Diastolic dip (%)	19.0 (11.5–26.7)	18.4 (11.0–25.8)	0.81
**Carotid intima-media thickness (mm)**	0.71 (0.59–0.82)	0.71 (0.61–0.82)	0.92
**Lipid profile**			
Total cholesterol (mmol/l)	4.11 (3.35–4.88)	4.02 (3.31–4.72)	0.74
LDL-C (mmol/l)	2.80 (2.09–3.51)	2.56 (1.91–3.21)	0.38
HDL-C (mmol/l)	1.05 (0.84–1.26)	1.01 (0.82–1.21)	0.65
Total cholesterol: HDL-C	3.98 (3.05–4.91)	4.13 (3.28–4.99)	0.66
Triglycerides (mmol/l)	0.78 (0.41–1.44)	0.93 (0.59–1.26)	0.29

## References

[b1] Caldwell J. C. & Schindlmayr T. Explanations of the fertility crisis in modern societies: A search for commonalities. Pop. Stud. 57, 241–263 (2003). 10.1080/0032472032000137790 14602528

[b2] Ayyavoo A., Savage T., Derraik J. G., Hofman P. L. & Cutfield W. S. First-born children have reduced insulin sensitivity and higher daytime blood pressure compared to later-born children. J. Clin. Endocrinol. Metab. 98, 1248–1253 (2013). 23365122 10.1210/jc.2012-3531

[b3] Ayyavoo A., Derraik J. G., Hofman P. L. & Cutfield W. S. Is being first-born another risk factor for metabolic and cardiovascular diseases? Future Cardiol. 9, 447–450 (2013). 23834681 10.2217/fca.13.41

[b4] Siervo M., Horta B. L., Stephan B. C., Victora C. G. & Wells J. C. First-borns carry a higher metabolic risk in early adulthood: evidence from a prospective cohort study. PLoS ONE 5, e13907 (2010). 21085691 10.1371/journal.pone.0013907 PMC2976719

[b5] Ong K. K., Preece M. A., Emmett P. M., Ahmed M. L. & Dunger D. B. Size at birth and early childhood growth in relation to maternal smoking, parity and infant breast-feeding: longitudinal birth cohort study and analysis. Pediatr. Res. 52, 863–867 (2002). 12438662 10.1203/00006450-200212000-00009

[b6] Savage T. *et al.* Birth order progressively affects childhood height. Clin. Endocrinol. (Oxf.) 79, 378–385 (2013). 10.1111/cen.12156 23347499

[b7] Jelenkovic A., Silventoinen K., Tynelius P., Myrskylä M. & Rasmussen F. Association of birth order with cardiovascular disease risk factors in young adulthood: a study of one million Swedish men. PLoS ONE 8, e63361 (2013). 23696817 10.1371/journal.pone.0063361 PMC3656047

[b8] de Bock M. *et al.* Olive (*Olea europaea* L.) leaf polyphenols improve insulin sensitivity in middle-aged overweight men: a randomized, placebo-controlled, crossover trial. PLoS ONE 8, e57622 (2013). 23516412 10.1371/journal.pone.0057622 PMC3596374

[b9] Matsuda M. & DeFronzo R. A. Insulin sensitivity indices obtained from oral glucose tolerance testing: comparison with the euglycemic insulin clamp. Diabetes Care 22, 1462–1470 (1999). 10480510 10.2337/diacare.22.9.1462

[b10] Lorenzo C., Haffner S. M., Stancakova A. & Laakso M. Relation of direct and surrogate measures of insulin resistance to cardiovascular risk factors in nondiabetic Finnish offspring of type 2 diabetic individuals. J. Clin. Endocrinol. Metab. 95, 5082–5090 (2010). 20702522 10.1210/jc.2010-1144

[b11] Maki K. C., Rains T. M., Dicklin M. R. & Bell M. Repeatability of indices of insulin sensitivity and secretion from standard liquid meal tests in subjects with type 2 diabetes mellitus or normal or impaired fasting glucose. Diabetes Technol Ther 12, 895–900 (2010). 20879960 10.1089/dia.2010.0083

[b12] Schutz Y., Kyle U. & Pichard C. Fat-free mass index and fat mass index percentiles in Caucasians aged 18–98 y. Int. J. Obes. Relat. Metab. Disord. 26, 953–960 (2002). 12080449 10.1038/sj.ijo.0802037

[b13] Casella I. B. *et al.* A practical protocol to measure common carotid artery intima-media thickness. Clinics 63, 515–520 (2008). 18719764 10.1590/S1807-59322008000400017 PMC2664129

[b14] Hagströmer M., Oja P. & Sjöström M. The International Physical Activity Questionnaire (IPAQ): a study of concurrent and construct validity. Public Health Nutr 9, 755–762 (2006). 16925881 10.1079/phn2005898

[b15] Facchini F. S., Hua N., Abbasi F. & Reaven G. M. Insulin resistance as a predictor of age-related diseases. J. Clin. Endocrinol. Metab. 86, 3574–3578 (2001). 11502781 10.1210/jcem.86.8.7763

[b16] Loos R. J. The metabolically healthy overweight and obese and their impact on all-cause mortality. Obesity 21, 1750–1752 (2013). 24078232 10.1002/oby.20601

[b17] Lopez-Garcia E., Guallar-Castillon P., Leon-Muñoz L. & Rodriguez-Artalejo F. Prevalence and determinants of metabolically healthy obesity in Spain. Atherosclerosis 231, 152–157 (2013). 24125427 10.1016/j.atherosclerosis.2013.09.003

[b18] Kilpeläinen T. O. *et al.* Genetic variation near IRS1 associates with reduced adiposity and an impaired metabolic profile. Nat. Genet. 43, 753–760 (2011). 21706003 10.1038/ng.866 PMC3262230

[b19] Robertson W. *et al.* The placental bed biopsy: review from three European centers. Am. J. Obstet. Gynecol. 155, 401–412 (1986). 3526901 10.1016/0002-9378(86)90843-4

[b20] Khong T. Y., Adema E. & Erwich J. On an anatomical basis for the increase in birth weight in second and subsequent born children. Placenta 24, 348–353 (2003). 12657508 10.1053/plac.2002.0922

[b21] Lang U. *et al.* Uterine blood flow–a determinant of fetal growth. Eur. J. Obstet. Gynecol. Reprod. Biol. 110, **Supplement**, S55–S61 (2003). 12965091 10.1016/s0301-2115(03)00173-8

[b22] Santulli G. & Iaccarino G. Pinpointing beta adrenergic receptor in ageing pathophysiology: victim or executioner? Evidence from crime scenes. Immun Ageing 10, 10 (2013). 23497413 10.1186/1742-4933-10-10 PMC3763845

[b23] Santulli G. *et al.* Age-related impairment in insulin release: the essential role of β_2_-adrenergic receptor. Diabetes 61, 692–701 (2012). 22315324 10.2337/db11-1027 PMC3282797

[b24] Savage T. *et al.* Increasing maternal age is associated with taller stature and reduced abdominal fat in their children. PLoS ONE 8, e58869 (2013). 23527040 10.1371/journal.pone.0058869 PMC3604016

